# What Can We Learn From Nurses' Experiences of Digital Technology Implementation During the COVID‐19 Pandemic? A Qualitative Study

**DOI:** 10.1111/jocn.70155

**Published:** 2025-12-10

**Authors:** Dawn Dowding, Louise Newbould, Nicholas R. Hardiker, Rebecca Randell, Manoj Mistry, Muhammad Faisal, Sarah Skyrme

**Affiliations:** ^1^ School of Health Sciences, University of Manchester Manchester UK; ^2^ Department for Transport, UK Government London UK; ^3^ School of Social Care Research, Church Lane Building, University of York Heslington UK; ^4^ School of Human & Health Sciences, University of Huddersfield Huddersfield UK; ^5^ Faculty of Health Studies, University of Bradford Bradford UK; ^6^ Wolfson Centre for Applied Health Research Bradford UK; ^7^ Patient and Public Contributor Manchester UK; ^8^ NIHR Yorkshire and Humber Patient Safety Research Centre (YH PSRC), Bradford Institute for Health Research Bradford UK; ^9^ Centre for Primary Care and Health Services Research, Institute of Population Health, University of Manchester Manchester UK

**Keywords:** COVID‐19, digital technology, nursing, qualitative research

## Abstract

**Aim:**

To explore nurses' experiences of the adoption, implementation, and use of digital technologies during the Covid‐19 pandemic in the UK.

**Design:**

A qualitative descriptive study.

**Methods:**

A qualitative study using two data sources: qualitative responses from 55 respondents to an online survey, and data from in‐depth interviews with 21 individuals. The NASSS framework was used to guide data collection and analysis. Data were analysed using framework analysis.

**Results:**

Respondents reported using a variety of technologies including video conferencing applications, telemonitoring, systems to support care management and telecommunication systems. The analysis identified a range of reasons why technology had been introduced into services, and a recognition of its value in a situation where otherwise care may not have been able to continue. During the pandemic nurses were expected to change their work practices very rapidly, and we identified situations where organisational infrastructure either supported this effectively or created additional burdens for the nurses' work.

**Conclusion:**

Nurses had to adapt to new ways of working rapidly, with digital technology being one of the primary means through which communication and care were delivered. The Covid‐19 pandemic provided a unique set of circumstances where layers of governance and many of the existing barriers to technology introduction were reduced.

**Implications for the Profession:**

It is important to learn from these experiences, to understand how to sustain innovations that have proved to be successful, as well as the factors that enable nurses to work effectively in this new environment.

**Reporting Method:**

This study adheres to the guidance for publishing qualitative research in informatics.

**Patient or Public Contribution:**

A public contributor was involved from the beginning of the study conceptualization. They had input into the study approach, were part of the team that acquired the funding for the study and gave input at various stages into the processes for data collection, analysis and writing up the findings. The public contributor is a co‐author on this paper and has been involved in the writing and editing of this report.

**Summary**

- Problem being addressed
  ○ The Covid‐19 pandemic led to the rapid adoption of multiple digital technologies to support nursing practice.
  ○ There is insufficient knowledge on the impact the adoption of such digital technologies has had on nurses' work.
- Study main findings
  ○ The pandemic created a unique environment where there was a convergence between the usefulness of digital tools to support work that otherwise could not have occurred, and the organisational impetus to support it.
- How this study might affect research, practice or policy
  ○ The facilitators of rapid technology adoption identified in this study can help inform future digital technology implementation and inform policy development to reduce organisational barriers to adoption.

## 1 Introduction

Healthcare technologies played a significant role in supporting patient care during the Covid‐19 pandemic, and the rapid adoption and adaptation of technology was vital for facilitating safe working practices that minimised in‐person contact (Chang et al. 2021; Greenhalgh, Wherton, et al. 2020). Covid‐19 has created multiple, rapid, real‐life implementations of digital technologies (Hawley‐Hague et al. 2024), and expedited innovations that have long been discussed (Collins 2020), but which required buy‐in from patients and clinicians. Nurses are pivotal to the delivery of effective health care (Abdolkhani et al. 2022), and the successful implementation of digital innovation is likely to necessitate some adaptations to their roles and the ways they work. This change can benefit from well‐informed support that ensures a good fit between technology, the user and the working environment (Dykes and Chu 2020). Prior to the Covid‐19 pandemic, there were numerous challenges to the implementation and adoption of digital technologies in the UK, with nurses reporting issues such as poor digital infrastructure, limited interoperability between systems and poor system usability (Royal College of Nursing 2018; Schoville 2009; Zuzelo et al. 2008).

## 2 Background

Despite the rapid adoption of technologies during the Covid‐19 pandemic, and the impact this has had on all health care practitioners, there is insufficient knowledge regarding nurses' experiences during this time period (Abdolkhani et al. 2022). A review of 21 studies found a lack of exploration of the impact on nursing practice (Abdolkhani et al. 2022). There were only four studies exploring nurses' experiences, and overall there was a lack of studies examining innovations other than teleconsultations (Abdolkhani et al. 2022). A survey of UK nurses found that technologies introduced during the Covid‐19 pandemic had a number of functions including e‐prescribing, online communication, patient monitoring/data sharing and virtual appointments (Dowding et al. 2023). Some of these technologies were already in use across health care services prior to the Covid‐19 pandemic; however, in the context of an emergency setting, they were adopted at speed, and often without support for, or consultation with nursing staff. Nurses frequently reported they had not received sufficient training to use the systems, and there were persistent issues such as inadequate IT infrastructure including poor connectivity and lack of devices, and a workforce with insufficient digital literacy (Dowding et al. 2023). However, it is clear that in many services the transformation of service provision supported by digital technology has been successful and will be an important part of how we deliver services in the future (Hutchings 2020).

Many of the initiatives introduced in the United Kingdom were on a short‐term basis to meet an immediate need; for example the use of ‘off the shelf’ products that providers initially supplied for free, but which may not be sustainable over time (Hutchings 2020). The adoption of technology is usually a process rather than a one‐off event (Greenhalgh et al. 2008), and despite the pressing conditions created by the Covid‐19 pandemic, the embedding of tools may require adjustments. Many nurses now have direct experience of the benefits and drawbacks of implementing digital technologies, and details of their specific contexts (Kaplan 2001) provide learning on adoption processes. This learning can help indicate some of the elements required for initiatives to be successfully adopted, sustained or scaled up across services. It is important to have an in‐depth understanding of what worked well for nurses, and of any challenges in the post Covid‐19 era as this will help inform decisions on the use of digital alternatives for health care delivery (Health Canada 2024) and the long‐term embedding of technology into clinical settings (Clarke‐Darrington et al. 2023).

The purpose of this study was to explore nurses' experiences of the adoption, implementation and use of digital technologies during the Covid‐19 pandemic in the UK.

## 3 Methods

The study aims were addressed using two data sources: (1) qualitative responses from an online survey (Dowding et al. 2023) and (2) data from in‐depth interviews.

### 3.1 Theoretical Framework

The non‐adoption, abandonment, scale‐up, spread and sustainability (NASSS) framework guided study design and analysis (Greenhalgh et al. 2017). NASSS considers seven domains of healthcare that contribute to the implementation of technology: the condition or illness; the technology; the value proposition; the adopter system of staff, patients, carers; the organisation; the wider context; embedding and adaptation over time (Greenhalgh et al. 2017).

### 3.2 Survey

The methods for recruitment, data collection and analysis of quantitative findings from the survey have been reported elsewhere (Dowding et al. 2023). Briefly, an online survey was developed using Qualtrics XM software to examine respondents' experiences of (up to three) digital technologies that had recently been adopted into their services, and a final optional section invited nurses to share their broader attitudes to healthcare technology. Survey participants were recruited via professional nursing networks and social media. The survey provided participants with the opportunity to complete free‐text responses; these data are reported in this paper. At the end of the survey nurses were asked if they would be interested in participating in an interview.

### 3.3 Interviews

#### 3.3.1 Recruitment

Nurses who completed the survey and gave consent to be contacted were invited to be interviewed. Additional recruitment was carried out via professional nursing networks and social media mechanisms, with nurses who were UK‐based and who had been using digital technologies for patient care during the Covid‐19 pandemic invited to contact the team. Once potential participants made contact, they were supplied with further details including a participant information sheet and consent form and were given the opportunity to ask questions before deciding if they wanted to take part. In total, 21 nurses were recruited, representing England, Wales and Northern Ireland. No nurses from Scotland responded to the call for participants.

#### 3.3.2 Data Collection

Semi‐structured interviews were undertaken by two researchers. Both researchers are experienced in qualitative interviewing, and one had experience exploring the impact of digital technologies in health care. Neither of the researchers is a nurse and had no prior relationship with any of the interviewees. The interviews lasted approximately 25 min to 1 h and took place from April to July 2022 using Zoom's video conferencing platform. The interview schedule drew from semi‐structured interview prompts based on guidance from NASSS interview tools (Greenhalgh, Maylor, et al. 2020) and was also informed by issues raised by respondents in the free‐text responses to the survey. The prompts were designed to cover each of the seven NASSS domains. Several iterations of the schedule were drafted with the support of the study team, with further adaptations added after the first three interviews, based on points raised by participants. The NASSS framework is a flexible tool that has enabled a responsive approach to the study that recognises the complex and diverse ways in which technologies are implemented. The domains support multifaceted inquiry as the ongoing viability of the technology is influenced by a shifting range of contextual factors encompassing staff and patients and embedding of technology over time (Greenhalgh, Maylor, et al. 2020). With the participants' consent, the interviews were audio recorded then transcribed and anonymized to remove identifiable details. All interview data were stored on a secure, two‐factor authentication university database.

#### 3.3.3 Data Analysis

Transcripts from the interviews were uploaded to NVivo 12 Plus software. Data from the interview transcripts and qualitative survey responses were analysed using a framework analysis that utilised the NASSS framework (Gale et al. 2013). One researcher (SS) undertook the analysis, with a sample of 2 transcripts additionally coded by a second researcher (LN). Initial codes were shared with the study team, which comprised health care researchers, digital nursing leads and a PPI representative, to discuss and agree on emerging codes and categories (Greenhalgh, Maylor, et al. 2020). This process continued across several meetings until a consensus was reached. The two researchers then finalised the organisation and application of the agreed codes to the NASSS framework, utilising these in the analysis of the transcripts.

#### 3.3.4 Ethical Considerations

The University of Manchester classified the survey as a service evaluation and therefore ethical approval was not required for that element of the study. Approval to conduct the current interview study was given by the University of Manchester Research Ethics Committee, approval reference 2021‐13085–21598.

## 4 Findings

### 4.1 Respondent Characteristics

There were 55 respondents to the survey, who reported on 85 separate technologies. Respondents were mainly from Acute Hospitals and reported their experiences with a variety of different types of technology, with the most common technologies being those that enabled communication/consultation and remote monitoring.

In total 21 individuals comprising 16 females and five males took part in interviews. The majority specialised in supporting the uptake of digital interventions for the health or social care service in which they worked (*n* = 8), primarily working for a digital team within a Health and Social Care or NHS Trust (*n* = 5), with one participant working across informatics and clinical education (*n* = 1). This was closely followed by those working within a community team (*n* = 4). Less well‐represented groups were those working in primary care (*n* = 1) or third sector organisations (*n* = 1); no midwives were represented.

Table 1 highlights the types of technologies discussed, the primary reason for their introduction, and the specialty in which they were implemented. Twenty‐five technologies were discussed over twenty interviews (one was a joint interview between two participants).

**TABLE 1:** Technologies discussed by interview participants.

Technology and purpose	Speciality
Videoconferencing (VC) Applications	
Virtual consultation & ward rounds	Social Care (connected to care homes)
Education social care staff	
Video conferencing group therapy (DBT)	Mental Health
Virtual outpatient clinics & end‐of‐life‐care	Palliative care
Nurse led clinics	Palliative Care/social care/Reablement
Daily team updates	Wound Care
Patient consultation	Adult and Children Services in the Community
Clinical education	Various
Patient consultation	Mental Health
Carer support (videoconferencing & store‐forward)	Dementia Care
Telemonitoring	
Monitor clinical symptoms remotely	COPD
Monitoring of wounds	Wound Care
Telemonitoring/surveillance	Mental Health (inpatient ward)
Care management	
Community nursing scheduling tool	Wound Care/Diabetes and Palliative Care
Handovers & care management	Paediatrics (expanded to ICU & other depts.)
Care management	Paediatrics
Inpatients bed management	Various
Electronic patient records	Various
E‐observations (e‐obs)	Entire Acute Trust
Improving consistency in information/e‐obs	Nursing and Midwifery
Telecommunication (audio/messaging only – use of phones, baby monitors)	
Community nursing appointments	Wound Care
Virtual desktop/team messaging	Older People Primary Care
Advice/assessments	Sexual Health
Staff support	District Nursing/Social Care/Palliative Care
Communication (nurses – patients)	Covid Ward

The synthesised findings from the free‐text responses to survey questions and our interviews are reported below according to the seven domains of NASSS: condition (or illness), technology, value proposition, adopter system, organisation(s), wider system, embedding and adapting over time. Quotations to support the results are in Table 2.

**TABLE 2:** Quotations to support NASSS domains of analysis.

NASSS domain of analysis	Quotations
Condition	Q1: ‘we actually had baby monitors in the room and then from outside we could communicate with patients in a really quick manner rather than putting on all our PPE. I think that meant that patients felt they had more contact with us during the day.’ [PN07] Q2: ‘Then when we dug a bit deeper into why people weren't keen on video conferencing, the two main things that kind of came out, some people had a paranoia being monitored, that came with their psychosis, so people being able to tap into them online and like different levels of that. So we had some people who had kind of felt that anyway, that there were cameras in the walls and stuff like that. Then people who just didn't quite have faith in the technology.’ [PN13] Q3: ‘a lot of the people in that group had a lot of high risks around suicide, self‐harm. So we wanted to continue anyway but we felt it was especially important just to try and do that as quickly as possible to keep, any sort of normality for people and that connection. So it was …used for that purpose anyway.’ [PN01] Q4: ‘I am concerned that remote/virtual clinics could miss something important as people behave differently when online compared to face to face. For long‐term, stable patients, I can see the value in virtual clinics as their face to face clinics are brief.’ [SURVEY]
Technology	Q5: ‘So, video consultation for example, I was aware of it, but it was something that was almost a theoretical thing that you never saw in practice, to becoming the mainstay of how we now actually interact, how we provide a lot of services.’ [PN04] Q6: ‘as the pandemic was going on we implemented loads of things, so there was … and … phone calls, text messages. We even uploaded videos, so going back to trying to explain to people why they couldn't go out, other people in the team sort of did like videos of explaining why you can't go out at the moment in a dementia friendly way and uploaded it on to a website so that carers could access that repeatedly, rather than them having to repeatedly explain to somebody. So, like I say it was one tool in among loads that we started using.’ [PN16] Q7: ‘The challenges around using a device in rural areas can often be the connectivity and that's a challenge… the iPads that we were using… It wasn't the same experience as using a PC entirely, your scroll functions and your sort of pinching.’ [PN19] Q8: ‘Working in rural areas highlighted lack of network connectivity for doing satellite work. Also highlights computer understandings within the team.’ [SURVEY]
Value proposition	Q9: ‘Because it all happened at the start of the pandemic and was put in very quickly, I don't think we had time to do a proper engagement piece, we didn't have time to do a proper education piece and it was all quite rushed.’ [PN15] Q10: ‘The model was just, you know, initially intended for use on a, you know on wards, so we hadn't kind of considered it from the perspective of ED particularly at that stage, although there was agreement when the fast roll out went, that it would go there, so it wasn't, it wasn't just thrust upon them but, I think quite quickly afterwards we realised that it wasn't working as well in those areas.’ [PN05] Q11: ‘So you know for some people it was fantastic because they didn't have to walk, you know, they didn't have to come to this hub anymore. They didn't have to sit in a reception and wait and there's lots of people around and that would make them feel very anxious. And you know, some of the people would have panic attacks and that kind of thing. Or they'd maybe be late because their buses were late and then they'd get really stressed out. So for some it was fantastic and they loved it.’ [PN01] Q12: ‘And it's like if a patient attends outpatients the clinician seeing them can actually look in their record and look at the previous, when they've been in the hospital, they can look at their letters, it links through to our pathology system so they can see the blood results, it links through to the X‐ray system so you can see scans and X‐rays that they've had, so you're getting a good, complete picture of the patient before they actually walk through the door rather than trawling through lots of paper notes that go missing or are written in different places.’ [PN03] Q13: [there are] lots of positives from new technologies, better more efficient ways of working but also an expectation that new ways of working are in some cases an addition to all the other normal ways of working… and are extra work to existing busy caseloads. [SURVEY]
Adopter system	Q14: ‘I think the big thing with Covid is that staff were constantly being asked to change everything.’ [PN17] Q15: ‘It's almost because we are trying to change the whole of the way we work in incremental steps, but as we are making that change, I think people are exhausted. They are, just too much happened in a very short space of time, too much change. Because there is a lot of things been thrown at them and having to work differently over the last two years’ [PN15] Q16: ‘One thing the pandemic threw up was… A lot of our middle to senior level staff went through university and nurse training… when everything was still on paper… And those people still have that phobia around using computers.’ [PN15] Q17: ‘So it's changed how we work massively, it's really, really—I was really, really reluctant and sceptical at first, I was quite vocal in being sceptical about it but I'm happy to have been proven wrong that actually it's been very beneficial it's made our job more accessible I think to clients which is a good thing’ [PN16] Q18: ‘From the patient's point of view, doing outpatient clinics, who is actually showing them what they have got to do or given them any guidance? All they got was a text message or an email, not helpful. And when a significant proportion of our population is ageing and frail and ill, you know, you can be any age and be ill, and just not be able to function the way you would normally if you were well.’ [PN15]
Organisation(s)	Q19: ‘For me it just completely brought into sharp relief how far behind my Trust is on digital maturity and the ability to implement digital technology in a safe and appropriate way.’ [PN04] Q20: ‘I do think there are significant pockets of people who have the vision and the capability and the drive to implement digital solutions. I think there is a divide between those people and your clinicians, your nurses… doing the patient care. And I am not saying they won't do it… they can if it's supported and implemented in the right way.’ [PN15] Q21: ‘Health informatics and digital health teams, [and] our education teams… all got told to work from home. …you have to be accessible, and we weren't.’ [PN15] Q22: ‘During the waves of Covid, the clinical staff, the operational management, weren't around to really focus on it’ [PN04]. Q23: The use of technology changing practice appears to be enforced in areas where the staff have little power (ward nurses, junior ward doctors) but less so in those able to dictate their work (research, research pharmacy, senior doctors doing clinics, independent services e.g., radiotherapy, triage). This uneven introduction harms patient care as data is not shared well from these powerful services, increases pressure on the low power services as they have to pilot much of the technology and can increase resentment across the hospital due to power dynamics. [SURVEY] Q24: ‘Actually there wasn't the right sort of senior leadership, no one seemed to be able to explain who was working day‐to‐day on the project, where this has come from, who has started it, who initiated it, who was the responsible person who made this happen’ [PN04] Q25: ‘I think the other thing that we've done during Covid is… virtual training sessions where we've sent out multiple dates that users have been then able to attend group training sessions… [and] videos that we're able to send out to teams… to ensure that they've got the knowledge and understanding of the functionality that's being released.’ [PN03]
Wider system	Q26: ‘Think that Covid has advanced the digital agenda in the NHS by probably 15 years. That's a number that's sort of been coming out, because the understanding of digital, so we normally have X amount of red tape to get through—Covid, that went out the window, we just cracked on and did it.’ [PN02] Q27: ‘Because people have become a lot more digitally literate over Covid you know, the general population. The majority of people now know how to use Zoom or FaceTime or Teams and that allows us to do a lot more digitally that they can engage with than maybe before.’ [PN07] Q28: ‘What I am most concerned about is that NHS technologies are not integrating. Ideally, an integrated patient record accessible across the country would be most useful. As it is, each Trust is adopting electronic recording technologies that do not connect and one of the biggest problems is cross‐Trust communication as it is! New tech should help to connect, not maintain borderlines.’ [SURVEY] Q29: ‘Our systems don't talk to one another. And it's been a long‐standing problem trying to get interoperability between technologies.’ [PN09]
Embedding and adaption over time	Q30: ‘But if we can see more patients quicker and more effectively by using the technology then I think we have got to embrace it really. And I think it's here to stay so either you move with it or you move away I think that's the only option really isn't it?’ [PN12] Q31: ‘And they think because we are kind of on the downside of the pandemic we can just go back to doing what we did before so we don't need to do that anymore, we don't need to use that technology anymore. Whereas they should be saying, this is the new way of working and we should be doing this.’ [PN15] Q32: ‘I think perhaps we do need a bit of time to [think], which bits were good, which bits not so good, how can we help embed them with the staff that weren't so quick to catch on, and yes, it's lovely to go quickly but sometimes it leaves people behind.’ [PN14] Q33: ‘We can be more creative and innovative moving forward, with blended learning, we can do a lot more, put on [a] bigger amount of, get to a lot more people in a shorter space of time.’ [PN06] Q34: ‘You can't do that engagement and education, that change management piece, working from your office bedroom can you? You have to be visible, you have to be accessible, and we weren't. And I think if it was to happen again, and we are already actually working on it, we are working on having easily accessible, visible space where staff can just come in and ask questions about digital solutions.’ [PN15]

### 4.2 NASS Domain of Analysis: Condition

Technologies such as remote conferencing for rehabilitation or support sessions, and remote patient monitoring tools were rapidly made available to individuals with a variety of clinical conditions (e.g., heart failure and mental health issues). The need to respond quickly to ensure patients had access to care meant that technical solutions addressing short‐term requirements were introduced (Q1). Respondents raised concerns about how vulnerable individuals, who may not have access to the internet, or who lacked the capability to use digital technologies were potentially affected by the move to digital care, with technology being a barrier to care for patients with certain conditions or specific circumstances (Q2). For patients undergoing therapy for mental health and addiction issues, going online was a solution that ensured they continued to get vital support, albeit as a temporary measure (Q3). There were concerns about the long‐term impacts of managing clinical consultations online for some groups of patients (Q4).

### 4.3 NASS Domain of Analysis: Technology

Digital technologies were crucial in maintaining clinical practice during the pandemic, which acted as an accelerant to the uptake of technologies that had already been available, if rarely used (Q5). It also provided a platform for the introduction of a variety of different tools and strategies for communication that were new to health and care organisations (Q6).

Our respondents highlighted issues with an existing infrastructure that was not able to support the innovations. These included problems with connectivity, digital literacy and the availability of suitable devices, which are significant factors in providing joined‐up, supportive care to patients and are also crucial to the successful adoption of technologies (Q7; Q8).

### 4.4 NASS Domain of Analysis: Value Proposition

Technologies are more likely to be adopted if they can provide value to suppliers of the technology/software, the organisations purchasing it, and to patient care and clinicians. For participants, technology solutions were adopted at pace, often without some of the processes and procedures that would normally be observed, and which were sacrificed for speed (Q9; Q10).

Despite the speed of adoption, and nurses' associated frustration with the perceived lack of input into how a technology is implemented and its functions, there were many examples of benefits to the adoption of technologies. These included being more convenient and a less stressful experience for some of the individuals receiving psychological care (Q11). There were examples of improved care workflows for clinicians and an increased ability to manage patients in the community in a more efficient and effective way (Q12). However, one drawback to innovation occurred when nurses who were adopting new technologies simultaneously had to follow previous processes of care management, resulting in an increased workload (Q13).

### 4.5 NASS Domain of Analysis: Adopter System

The constant level of adoption and change to working practices meant staff had to adapt and learn new skills, often challenging their existing clinical practices (Q14; Q15). The new way of working that was necessitated by the pandemic foregrounded the need for digital literacy among the nursing workforce (Q16). Many of our respondents indicated there was more acceptance of digital technologies to support care, and the benefits were being seen in the way they work and in the care of their patients, despite initial skepticism (Q17). Patients, their families, and carers have also had to change the way that they interact with health and care services. Many of the individuals cared for by nurses in our interviews had developed skills in using digital technologies and embraced initiatives such as remote consultations and monitoring. However, in some cases, technology was seen as a barrier to care, and without sufficient support, or traditional options for care delivery, some patients could be excluded (Q18).

### 4.6 NASSS Domain of Analysis: Organisation(s)

The largely unanticipated changes in care provision using technology occurred irrespective of an organisation's readiness to change or their capacity for innovation. Among some respondents' organisations this caused problems, as they did not have the strategic infrastructure to support the adoption of digital technologies effectively and safely. In addition, respondents highlighted how readiness for change and the ability to embrace innovation varied within organisations and were often located with specific individuals rather than as a system‐wide strategy (Q19; Q20). One of the key factors to enable organisational success for the implementation of digital technologies is the resources available to support the work required. While organisations were expected to innovate at pace, the operational assets in terms of the staff and support required were not always readily available. A number of respondents highlighted that key operational staff were working at home and were not accessible on the front‐line to support staff in the way they normally would (Q21; Q22).

There was also concern about how decisions were taken at the organisational level, that then impacted on front‐line clinical staff who had little say in either the choice of technology being implemented or how it might work in practice. This could be seen in terms of hierarchies of power in organisations and was also attributed to a lack of expertise and leadership in informatics, as well as a lack of understanding that this may be important (Q23; Q24).

However, some had responded effectively to emergent needs, developing methods for supporting the implementation of technologies, including the provision of remote training and education (Q25).

### 4.7 NASSS Domain of Analysis: Wider System

The Covid‐19 pandemic was considered among several of our respondents to have accelerated the adoption of technologies beyond anything that would have been thought possible previously. In addition, the wider population (which includes health care staff, patients and the public) was perceived to have become much more digitally literate (Q26; Q27).

Respondents also highlighted wider contextual issues that remain, which will have an impact on the sustainability and spread of technologies post‐pandemic. This includes ongoing issues with the lack of interoperability of digital systems and the consequent difficulties in sharing and using data across organisations (Q28; Q29).

### 4.8 NASSS Domain of Analysis: Embedding and Adaptation Over Time

One key issue discussed by respondents was if and how the innovations will be sustained, and what factors may need to be considered in the longer term to support their continued use. Recognising the benefits of digital working was often seen as a driver to maintaining services digitally post‐pandemic (Q30). However, there was also acknowledgment that some staff may want to return to their previous ways of working (Q31). One respondent highlighted how there needs to be a period of reflection, to identify what strategies worked for implementation and how to revisit things to make sure staff are not left behind (Q32). A number of respondents discussed how their approaches to education and training of staff also had to change rapidly, with a move to more online education, and provision of support in new and innovative ways. Moving forwards there was both a recognition that some of this may need to be revisited, to sustain the changes already made, as well as making use of the new approaches to training as a standard for the future (Q33; Q34). In the event of a future health emergency, readiness to cope efficiently and with a cohesive approach within and across organisations is vital in safeguarding staff and patients' wellbeing.

## 5 Discussion

The aim of this study was to explore in detail nurses' experiences of implementing digital technologies during the Covid‐19 pandemic. Most nurses who responded to our interview requests work to support digital interventions in acute hospitals and there was limited geographical spread across the UK, meaning our findings may only apply to specific contexts of technology adoption. Nevertheless, their contributions highlight diverse issues that are likely to remain salient if, as intended, healthcare in the UK moves from analogue to digital provision of services (Morris et al. 2025). Successful adoption and subsequent sustainability and spread of digital technologies rely on a complex interplay of factors (Greenhalgh et al. 2017) including the individuals using the technology, the system where it is introduced and the wider context. The Covid‐19 pandemic provided unique circumstances where layers of governance and many of the existing barriers to technology introduction were rapidly overcome (Kuijper et al. 2022). This rapid response and the benefits associated with the adoption of technology have been identified across health care settings including General Practice and in mental health (Konteh et al. 2022; Li et al. 2022). In some cases, it encouraged individuals to be more open to the use of technology as a tool for innovation, and for change more generally, with benefits to patients in terms of convenience, safety, and a better use of resources. However, challenges also remain; whilst many staff have embraced new ways of working, the findings from our survey and interviews suggest there may still be issues with the digital literacy of staff and patients, and issues of accessibility to technical infrastructure that is fit for purpose for patients and nursing staff. Our survey of nurses' use of digital technology during the Covid‐19 pandemic highlighted the variability in usability of technologies. It rated digital technologies using the SUS (System Usability Scale), with some technologies scoring as low as 20 (electronic patient records) and 37.5 (online communication). These SUS scores indicate the poor ‘fit’ of some technology systems; none of the systems had an SUS score > 70 (the industry standard for acceptable usability) (Dowding et al. 2023).

The NASSS framework highlights the importance of a variety of individual and system/organisation factors that need to be in place to enable effective adoption of technology (Greenhalgh et al. 2017). The pandemic created a unique environment where there appears to have been a convergence between the value proposition or usefulness of digital tools to support work that otherwise could not have occurred, and the organisational impetus to support it. Nurses were empowered to provide ongoing involvement and input, to ensure technology is a good fit for their patients' needs and those of their service. The rapid change that ensued during the pandemic helped to innovate vital care, which in some cases entailed minimal red tape. Nurses explained to us how they had to work flexibly and often with limited oversight in an emergency setting; an experience recounted by nurses and other health care professionals both nationally and internationally (Baird and Maguire 2021; Pahuja and Wojcikewych 2021; Sessions et al. 2021). High quality, real‐time technical support and coaching can reduce anxiety and conflicts arising from the adoption process (Borges do Nascimento et al. 2023). For nurses to develop digital competencies, they must feel encouraged to ask questions and explore how the use of technologies applies in their role with their patients (Clarke‐Darrington et al. 2023). This iterative process upskills staff to act as champions of effective technology use in a rapidly evolving digitised health system (Clarke‐Darrington et al. 2023).

While the issues raised in this article occurred during the pandemic, they have wider implications post Covid‐19 regarding the role of digital technology. The urgency of the pandemic revealed inherent weaknesses in some implementations, which lacked sufficient planning for integration into systems, amid limited consultation with nurses. Given plans for the future embedding of technology (Morris et al. 2025), nurses require real‐time technical support, better integrated technologies and more cooperation within and across services to facilitate implementations that ensure safe and beneficial patient care.

### 5.1 Limitations

Nurses who have experienced positive implementations may have been motivated to take part in the interviews; conversely, those who had negative experiences during implementation might have chosen to participate to share their concerns and perspectives. Due to the methods of recruitment, it is possible that more senior staff responded to the call for participants, and that more digitally literate staff saw the call on social media sites. Despite several efforts to achieve geographical representation from across the UK, no respondents from Scotland were recruited to the study.

## 6 Conclusion

The Covid‐19 pandemic was a time of immense challenges in healthcare delivery that necessitated the rapid adoption of new technologies across organisations. This study provides important insights for future emergencies by highlighting how despite ongoing issues with technology infrastructure and data sharing, organisations that embrace innovation and creativity, supporting staff through education and training, can introduce new ways of working to support new approaches to care.

## Author Contributions

**Dawn Dowding:** conceptualization, formal analysis, funding acquisition, methodology, writing – original draft; **Louise Newbould:** conceptualization, formal analysis, investigation, methodology, writing – review and editing; **Nicholas R. Hardiker:** conceptualization, funding acquisition, methodology, writing – review and editing; **Rebecca Randell:** conceptualization, funding acquisition, methodology, writing – review and editing; **Manoj Mistry:** conceptualization, funding acquisition, writing – review and editing; **Muhammad Faisal:** conceptualization, funding acquisition, methodology, writing – review and editing; **Sarah Skyrme:** methodology, project administration, formal analysis, investigation, writing – original draft.

## Funding

This work was supported by Burdett Trust for Nursing.

## Conflicts of Interest

The authors declare no conflicts of interest.

## Supporting information


**Data S1:** jocn70155‐sup‐0001‐supinfo.docx.

## Data Availability

The data that support the findings of this study are available on request from the corresponding author. The data are not publicly available due to privacy or ethical restrictions.
