# Supplementary material for: What Can We Learn From Nurses' Experiences of Digital Technology Implementation During the COVID‐19 Pandemic? A Qualitative Study
Source: J Clin Nurs. 2025 Dec 10;35(4):1797–806. doi: 10.1111/jocn.70155 (PMC12964503; doi:10.1111/jocn.70155)
Supplement: Supplementary file 1 — Data S1: jocn70155‐sup‐0001‐supinfo.docx. [file JOCN-35-1797-s001.docx]

**Guidance for reporting qualitative research in informatics**

1. Theory
   1. Cite theory appropriate to the topic being studied if applicable

*NASSS theory cited (p. 6)*

1. Research question and study design
   1. State the research question

*Research question cited (p.5)*

- 1. State the study design and methodological perspective of the research

*Study design and methodology described on p.6*

1. Sampling
   1. Describe the sampling approach

*Sampling approach described on p. 6-7*

- 1. Describe any approaches to ensure the inclusion of people from marginalized or underserved groups

*Study was focused on nurses working in the UK*

- 1. Report and justify the sample size

*Sample size reported (p. 6)*

- 1. If using saturation to determine sample size, report what type of saturation was used, and how saturation was assessed*

*N/A*

1. Data collection
   1. Report how data were collected

*Detail on data collection methods provided on p. 6-7*

- 1. Report any methods for reducing bias in data collection and analysis*

*N/A*

1. Data analysis
   1. Describe data analysis methods, with appropriate citations*
      1. For deductive analysis, report how the theory was used in the data collection and analysis*
      2. For inductive analysis, report how the steps of inductive analysis were done*
      3. For theory development, report how categories were developed*
   2. Describe any methods for improving the dependability of coding*
   3. Report any measures for improving the credibility of findings or verifying interpretations*

*Approach to data analysis, using a framework approach is detailed on p. 7-8, including how codes were used and process for improving dependability of the coding*

1. Results
   1. Report sample size and characteristics of participants

*Sample size and participant characteristics reported on p. 8-9*

- 1. Support thematic findings with extracts, quotes, images, or observations
  2. Provide synthesis and interpretation

*Thematic findings and synthesis/interpretation with data quotations is reported on p. 10-17*

1. Discussion
   1. Describe assumptions of the research and details of setting and context to illustrate transferability of findings
   2. Describe relationship of findings, or new theory developed in the study, to existing theory
   3. Report limitations

*All above issues reported in discussion where relevant on p. 18-19*
